# Understanding burnout in Pediatric residency through the lens of the ‘Areas of worklife’

**DOI:** 10.1080/10872981.2022.2152495

**Published:** 2022-12-06

**Authors:** Linessa M. Zuniga, Julieana Nichols, Teri Turner, Carla Falco

**Affiliations:** Academic General Pediatrics, Baylor College of Medicine and Texas Children’s Hospital, Houston, TX, USA

**Keywords:** Burnout, worklife areas, residency, medical education, autonomy

## Abstract

**Background and Objectives:**

Burnout is a widespread problem in medicine, especially among trainees. Despite this, data on effective interventions are limited. An organizational context for burnout entitled *Areas of Worklife* identified six areas of the work environment that can affect burnout through mismatches between individuals expectations of that area and the reality of the work environment. This study aimed to gain a deeper understanding of pediatric residents’ perspectives of the *Areas of Worklife* to allow programs to utilize this framework in the development of future interventions.

**Methods:**

Using qualitative methodology founded in grounded theory, we employed an iterative data collection by conducting semi-structured interviews, until data saturation was achieved, with 15 pediatric residents in 2018. We recorded interviews and transcribed them verbatim. Content analysis was conducted concurrently with data collection using constant comparison methods; the principal investigator and co-investigators worked jointly to generate codes and identify themes.

**Results:**

Themes were identified for the individual *Areas of Worklife* that represented resident perspectives and mismatches with the work environment. Overall, patient care was a central focus connecting the areas of control, reward, values, and workload; themes in these areas concentrated on resident’s ability to interact with and learn from patients.

**Conclusions:**

Residents’ definitions of the *Areas of Worklife* can be used to identify mismatches between residents’ expectations and their work environment, which can inform organizational interventions. These findings highlight the importance of a patient-focused approach to residency training, which is consistent with literature that shows patient care is a means to find meaning in their work. Resident definitions of the *Areas of Worklife* offer residency programs a practical approach in their battle against burnout by providing focused direction to respond to resident needs and identify tangible targets for intervention.

## Introduction

As literature on burnout skyrockets, its impact is exhibited in the negative provider attitudes and behaviors towards patients [1], increased medical errors [2], and its effect on individual happiness and work satisfaction[3]. Additionally, this boom in literature has partially revealed the why behind burnout and its complex interplay of systemic and interpersonal factors [4,5]. What is lacking, however, is a systematic methodology for implementing what we know to combat burnout efficiently and holistically.

A systematic methodology requires an understanding of the needs of those experiencing burnout vis-a-vis the reality of their work environment. Resident perspectives on the mismatch between their needs to prevent burnout and their work environment is lacking. Ironside, et al. examined residents’ and faculty’s perspectives on resident burnout and identified 5 themes: fatigue, cultural norms in medicine, the learning curve from medical school to residency, social relationships, and finding meaning at work[4]. Mylod proposed that a balance of inherent and external rewards and stresses can prevent burnout[5]. Maslach and Leiter approach workplace burnout through a comprehensive model titled the *Areas of Worklife*. This model identifies 6 *Areas* where potential mismatches between an individual’s expectations and the realities of their work environment affect burnout; the model encompasses previously identified factors that contribute to burnout and can be a roadmap as part of a systematic approach to combat burnout[6].

The work environment of residency is a unique blend of education and work. Before utilizing the *Areas of Worklife* to address burnout in this environment, it is critical to understand how these *Areas* apply since the model was not developed for healthcare or training environments. For example, according to the *Areas of Worklife*, a lack of control in the workplace leads to burnout, but what does control mean to a resident? Our study aims to create a foundational understanding of the *Areas of Worklife* in residency by exploring residents’ perceptions of the 6 *Areas* through qualitative methodology. These distinctive interpretations may help inform future use of this comprehensive model in similar residency programs to combat burnout.

## Methods

### Study design

Using qualitative methodology founded in grounded theory, we employed an iterative data collection and analysis process with content analysis using a constant comparison approach [7,8]. The 6 *Areas of Worklife* served as an organizing framework for data collection and analysis, including: 1) control, 2) values, 3) reward, 4) fairness, 5) workload, and 6) community[6]. The definitions for the *Areas of Worklife* as described by Leiter and Maslach can be found in Table 1. Additionally, mismatches were identified through questions that asked how the program could improve within the 6 *Areas* individually and overall whether the program was providing the training residents hoped for (see supplemental interview guide).

**Table 1.** Areas of worklife definitions.

Areas of Worklife	Definition
Control	includes individual autonomy and the ability to influence decisions affecting one’s work^6^.
Values	“encompasses the ideals and motivations” that originally attracted an individual to their job^6^.
Reward	“addresses the extent to which rewards- monetary, social, and intrinsic – are consistent with expectations.”^6^
Fairness	can be seen as whether “decisions at work are perceived as being fair and people are treated with respect.”^6^
Workload	can be seen as both qualitative and/or quantitative and is essentially the demands of an individual’s job^6^.
Community	defined as “the overall quality of social interaction at work.”^6^

### Context and participants

The setting was an urban, quaternary children’s hospital that houses the largest pediatric residency program in the country and includes tracks for global health, physician-scientist, and primary care/advocacy. Ongoing efforts to address burnout in the residency program focused on individual surveillance and interventions as well as system-level changes, including resident self-reflections, recurrent protected social time during the work day, the creation of a resident-led support team, resident appreciation week, and schedule adjustments with a transition to a predominantly night float system and patient caps on clinical, inpatient teams. Burnout rates at our institution have mirrored national rates with 50% of our upper level residents endorsing burnout at the time of the study.

### Interviews

Using qualitative methodology, the primary investigator (LZ) conducted semi-structured interviews with residents. Known to some participants as a former chief resident, during the study she had no involvement with the residency leadership team and was not involved in the evaluation of residents in any way. We used both a purposeful and convenience sampling approach by inviting residents at all levels of training during a non-clinical rotation to participate from January to June of 2018[9]. This timeframe ensured the participants had sufficient experience in residency. Approximately the same number of residents were approached for each year of training. A total of 15 residents participated: 2 interns, 2 second-years, 9 third-years, and 2 fourth-years.

Leiter and Maslach’s *Areas of Worklife* served as the framework for developing questions to elucidate perceptions of the *Areas* in the context of residency and ways to improve through specific interventions. The questions were developed jointly by the PI and co-investigators. [See supplement for interview guide].

Prior to interviews, residents received a brief introduction to the *Areas of Worklife* framework and its relationship to burnout from the interviewer (LZ). The introduction consisted of a short explanation as to what the *Areas of Worklife* was, the purpose of its creation, and its relationship to burnout and the interview. Of note, fairness and workload were added to the interview questions iteratively because they initially were considered to have straightforward definitions. However, after initial interviews uncovered the need for further exploration, questions about workload and fairness were added for the fifth interview onward. Interviews averaged 37 minutes, were recorded and transcribed verbatim. The Baylor College of Medicine IRB approved this study.

### Data analysis

We conducted content analysis concurrently with data collection, which is in line with grounded theory’s constant comparison approach[7]. We refined the codes using ATLAS.ti software (atlasti.com) to create themes. Interviews were conducted until data saturation was achieved, including for the themes of workload and fairness despite fewer interviews. To increase the trustworthiness [10] and strength of the study’s findings, two of the co-investigators independently coded a subset of transcripts; the codes were then discussed to establish a consensus. Peer debriefing was ongoing, with regular meetings to review the organization of codes and themes. In addition, member checking was performed with residents not part of the study, chief residents, and program leadership.

## Results

**Figure 1.**
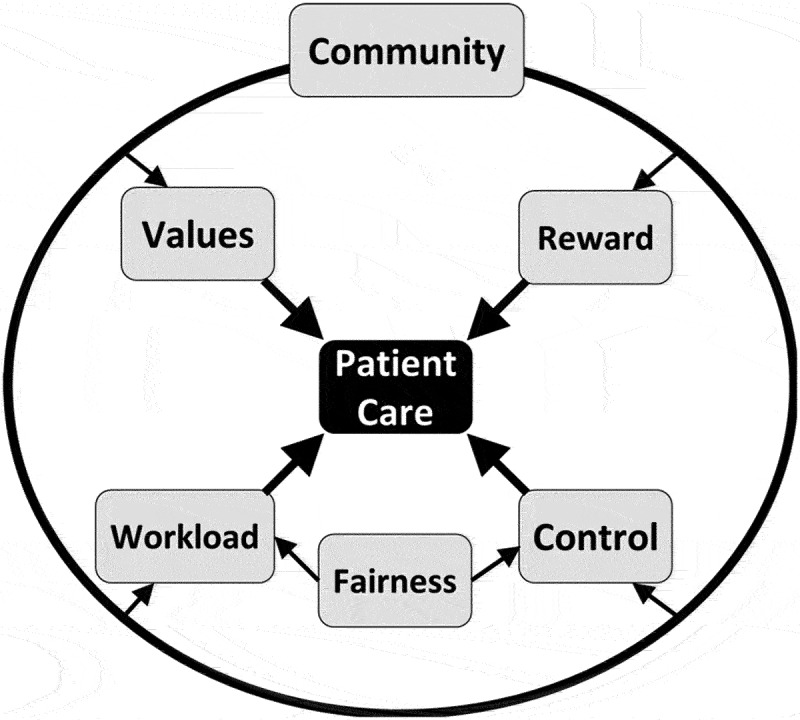
Residency *areas of worklife*: connectedness through patient care.

Major themes quickly emerged with robust descriptions of each *Area of Worklife* in the context of residency and identification of mismatches between the ideal presented by the resident and the actual work environment of the program (referred to as mismatches). One to 2 themes were identified for each area except for community, which had themes for the 5 relationships between residents and workplace community members. Table 2 contains illustrative quotes for each theme. Through the process of generating themes, the centrality of the patient was identified as a connecting theme for the areas of control, value, reward, and workload (Figure 1).

**Table 2.** Themes and representative quotes.

Control	Schedule		‘I think having control over your work means you know your schedule and you can help control the schedule. You know what time you’re gonna be rounding and you get to divvy up the patients and what patients you’re gonna see … ’
			‘At least getting to put in what most important day you want off or just the important things … that month to make sure you can be able to go to for mental wellness’
	Autonomy in Patient-Care Decisions		‘Autonomy gives you a sense of purpose. That you are making decisions, growing as a clinician. There’s going to be so many things outside of your control, but if you’re allowed to have control, safely, within your realm of what’s expected and safe for the patient so that you can grow, I think that’s important.’
Values	Learning	Patient Care	‘I think as values for us we get more out of actual patient care and the learning process that goes along the way … ’
			‘I value providing optimal care for patients, spending time with patients, and trying to learn from them.’
		Peer-to-Peer	‘You had a resident work room with other third years … People came in with a lot of different ideas, and you could bounce things off each other.’
Reward	Helping sick children get well		‘Well there’s the intrinsic rewards, right, of feeling like what we’re doing is actually contributing value to somebodies life or to some family and that’s always kind of irreplaceable if you actually feel like you’re making an impact’
	Recognition/Appreciation		‘Feeling appreciated like you did something that was helpful … or just feeling kind of recognized’
Fairness	Scheduling Equity		‘Yeah. I guess for me, the idea of fairness is … the sense that there is no exceptional treatment, that everyone is within the range of a certain expectation, like getting more or less the same thing out of the experience’
Workload	Recovery Time		‘ – it’s the relentless nature of back to back to back. I go through periods of months where I’m like, “I have not had a single day off.” Then they’re like, “Well, that’s not true. You had four.” I’m like, “Well, that was just recovering from – ” You know what I mean? If there was a way to have, during the really intense months, an actual, just a little bit of time where you don’t need to just be home … ’
	Non-educational Tasks		‘I don’t mind doing medical work, and I find that rewarding … what I don’t like about it is, fielding 1,000 phone calls, and changing this order or changing that order. That’s work that is just frustrating.’
Community	Nursing	Collaborative	‘At its best, it is collaborative and everyone recognizes that they’re on the same team.’
		Lack of Awareness	‘Maybe educating, nurses knowing when a resident is working might help because a lot of tension between residents and nurses comes when a nurse will call at 3:00 in the morning to ask about a clarification when a resident is on a 28-hour shift and that is their only hour to sleep’
		Increased interaction	‘we try to do nurse included rounds and I think those are really good but it’s still only a few minutes a day and it’s not really a conversation … but If there was some opportunity to have conversations in a group … just all sit and have lunch together, that might be interesting.’
			‘Maybe even doing more whole floor events, or lunches for the nurses and residents, or things that could help build relationships more’
	Advanced Practice Providers	Helpful Resource	‘They are fantastic. They help you out. When we have questions, they’re a great resource.’
		Lack Camaraderie	‘Separate. Isolated. Even when we are on the same team, it’s just very-they have their patients; we have our patients.’
		Increased Interaction	‘More interactions, I think it’s just getting to know people. Just working with them daily is gonna bring more of like a friendship’
	Attendings	Absence of Hierarchy	‘It’s not as hierarchical here, and so I think, in general, their relationship is very positive. You see your attending as a teacher, but also as a role model and as an ally.’
		Teaching	‘I think we have, for the most part, really fantastic attendings who like teaching.’
			‘I think to me what makes a good attending is that they support resident autonomy, they value education and teaching, they respect resident’s time, and they aren’t above offering to help and chip in.’
	Leadership	Supportive	‘I feel like they would go the extra mile to help a resident out’
		More Time	‘It might be helpful to be able to meet with more program leadership to talk about issues.’
	Residents	Biggest Support	‘I think that the other residents are probably our best resource as far as helping sort of cope and manage the difficulties of residency because they are some of the only people that have an understanding of the situations we are going through.’
		More Time	‘I don’t think we have enough time or opportunity to see the other residents.’

### Control

Two themes emerged when residents were asked about ‘control’ in residency: control over their schedules and control over patient-care decisions (i.e., autonomy in clinical decision-making). Residents cited the importance of schedule control from both macro and micro perspectives: requesting vacation blocks and days off, choosing the timing of rotations versus a desire to control time during the day and how to prioritize tasks. To correct mismatches in this area, residents suggested more freedom to decide which educational activities to attend (i.e., morning report and noon conference) as well as having more influence over the number and division of patients and structure of rounds. Additional suggestions for improvements included timing of schedule release, with earlier preferred, but with the caveat that daily schedules should be flexible (e.g., changing days off or arranging an unplanned late arrival/early departure) to accommodate spontaneous life demands without oversight from an authority figure.

Second, residents stressed autonomy in clinical decision-making as an important element of control. This was also an area of mismatch as many residents described the complexity of being allowed to make patient care decisions. Suggestions for improvement focused on intra-team communication with some decision making occurring out of the presence of the patient to maintain the trainee-patient relationship and clear expectations for the different team member roles. Finally, having the attending prompt and guide clinical decision making when necessary was particularly impactful as it demonstrated confidence in their development as clinicians.

### Values

Residents most valued learning, particularly related to patient care. They stressed the importance of spending time with patients, both to learn and develop the physician-patient relationship, and they distinguished between learning (education and being with patients) and clerical work (writing notes, placing orders, or performing other tasks that they perceived as being non-educational). Clerical work detracted from preferred time with patients. Residents noted a mismatch when they described how education was often sacrificed for the sake of efficiency. One suggested intervention included increased support on busy services (either through care/discharge coordinators or attending only teams) to allow for more time dedicated to bedside teaching, individual learning and reflection. Additionally, scheduling large blocks of time for didactics to protect from inpatient work was another suggestion. Lastly, residents cited learning from their peers as a meaningful experience as it was a low-pressure, safe form of education.

### Reward

Reward for residents focused on the extrinsic rewards of recognition and appreciation and on the intrinsic reward of helping sick children get well. Though extrinsic rewards did not reveal mismatches, descriptions varied and ranged from simple verbal praise to small acts of appreciation on the part of the program or hospital, such as providing meals. Recognition in the form of awards of excellence, such as intern/resident of the month, were also mentioned through to a lesser degree.

Many residents described the intrinsic reward of personally providing patient care and helping sick children. Witnessing an improvement in a patient’s illness and contributing to the benefit of the patient gave residents a sense of fulfillment and meaning in their work. ‘I find the work really rewarding … I like being able to see from admission to discharge that I’ve made an impact.’ Residents in general experienced this intrinsic reward, but some saw it as a mismatch because they desired more time at the bedside with patients and suggested increased support for clerical work.

### Fairness

The majority of residents identified fairness as scheduling equity, which most defined as having the same number of days worked/off, absence of favoritism, and equal treatment relative to other residents. This was not found to generally be an area of mismatch. Still, several residents suggested to improve fairness through increased transparency, which would include more information on how schedules are created and the decision process around scheduling requests and backup shifts.

In direct contrast to across the board equal treatment, one resident notably defined schedule equity as based on work performance. Specifically, the resident offered that more hard-working residents (e.g., did not miss days often and had good performance as demonstrated by evaluations) should receive a more favorable schedule.

### Workload

Workload themes emerged as ‘non-educational tasks’, or clerical work, and the importance of recovery time. Residents described their workload as including two components: medical decision-making and clerical work, such as changing orders. More medical decision-making was desirable and lessening clerical work was a key driver to improving workload. The electronic medical record process of admitting and discharging patients on high-turnover services was described as particularly taxing. Clerical work, as noted above, was found to be a mismatch for residents, and suggested interventions overlapped with those for the *Area* of value (see above). In contrast, when residents were actively engaged in medical decision-making, they could function within a high-intensity workload environment for a longer period of time: ‘When I go to the ER, and I work my butt off, I leave energized. I don’t leave depleted.’

Second, recovery time was described as sufficient time without work to allow the resident to recharge before returning to work. The description of sufficient recovery time varied among residents and depended on the rotation. Recovery time was not consistently reported as a mismatch; yet, they did recommend minimizing back-to-back, clinically demanding rotations, such as call months, and to provide, within the workday, protected time that allowed residents to decompress.

### Community

Finally, residents discussed their relationships with different groups within the healthcare community.

### Nursing and advance practice providers

Most respondents described nursing staff and APPs as friendly and collaborative, especially when initiated by the resident. However, these relationships were an area of mismatch. Suggested interventions included increasing awareness of the residents’ schedules since one resident recounted that explaining his/her schedule with a nurse resulted in greater collaboration and teamwork. The resident-APP relationship had contrasting facets with APPs seen as great resources while some felt a lack of camaraderie. This gap manifested itself in issues with division of workload and approachability. Suggested interventions to improve both relationships included increased interaction in a less formal context, such as outside of rounds, to bolster a sense of community.

### Attendings

Absence of hierarchy was the main characterization of the residents’ relationships with attendings and was not a mismatch. Attendings emphasized mutual respect, valued the work residents do, and made an effort to get to know the residents personally. Highlighting the importance of education, residents appreciated attendings who were humble and willing to teach and learn with residents.

### Residency program leadership

Residency program leadership included the physician-in-chief, program director, associate directors, and chief residents. This was not an area of mismatch as residents emphasized leadership support that manifested as resident comfort in approaching leadership with concerns or questions, specifically the chief residents and program director, and leadership being responsive in enacting necessary change.

### Among residents

Finally, and most notably, residents enthusiastically described relationships with each other as their main support. While not consistently a mismatch, many residents described a desire for more time to socialize in both small and large group settings, like intern retreat. Most residents relied greatly on each other in their hospital work and in coping with the challenges of residency. Several residents mentioned a like-mindedness when it came to work ethic and a team mentality.

## Discussion

Exploring Leiter and Maslach’s 6 *Areas of Worklife* from the resident’s perspective has not been studied previously and assessing these areas in the unique work environment of residency provides a deeper understanding of the applicability of this framework to combat burnout. We identified mismatches between what residents described and what existed in the work environment. Our results align with previous work that showed that residents have experienced a general decline in autonomy with an increase in clerical work [11] while maintaining little control over their general schedule, which increases burnout[12]. A robust community to prevent burnout, which has been emphasized by the COVID-19 pandemic, is also gaining traction [4,13]. Our study reveals the connection between fairness and scheduling equity for residents. Increasing scheduling transparency in the business world has improved satisfaction [14]. Based on these findings, we suggest several interventions that we hypothesize will combat burnout (Table 3). In addition, the connectedness between the 4 *Areas* of control, reward, values, and workload through a common theme of patient care (Figure 1) has not been previously described.

**Table 3.** *Areas of worklife*, mismatches, and proposed solutions.

Area of Worklife	Resident Perception	Mismatch	Proposed solution
*Control*	Control over schedule	General lack of control over resident schedules.	Increase schedule flexibility
			Provide more choice in the structure of the work day or rotation
	Control regarding decisions related to patient care.	Lack of autonomy over patient care decisions	Train the teams in communication Set expectations Conduct discussions on rounds outside the patient room
*Value*	Patient related and focused education	Clerical work taking away from learning, dedicated teaching time often interrupted	Engage care/discharge coordinators Protect teaching/didactic time in larger chunks to eliminate interruptions and task shifting
*Reward*	Extrinsic – Recognition and appreciation	None	Give regular verbal praise and acts of appreciation from the program or hospital
	Intrinsic – Helping sick children	Inability to witness this by lack of time at bedside, increased clerical work	See value solution* Enhance patient follow-up through established avenues (continuity clinic)
*Fairness*	Scheduling equity	None	Make scheduling process transparent
*Workload*	Clerical work	Negative contributor to workload	See value solution* Engage residents in medical decision making to offset clerical work
	Recovery time	None	Minimize back to back call months Protect time during the work day to decompress
*Community*	Collaboration with nursing	Lack of nursing awareness of resident schedule	Increase communication and interaction with nursing colleagues
	Camaraderie with Advanced Practice Providers (APPs)	Lack of camaraderie	Increased informal interaction with APPs
	Absence of hierarchy among attendings	None	Get to know residents personally Foster mutual respect
	Approachability of program leadership	None	Leadership is receptive to change Be approachable if residents have questions or concerns
	Residents as main form of support	None	Organize class events

Residents cited the importance of patient care in regards to **control** through autonomy in patient-care decisions, to **value** through learning via patient care, to **reward** through seeing the impact of resident’s care on patient outcomes, and to **workload** through having the time/ability to develop meaningful patient relationships. Our results support past studies that identified patient care as a critical element to finding meaning in work, which is correlated to less burnout [4,15–19]. Hipp et al identified the means to enhance meaning in work as ‘more time spent at the bedside with patients, with more engagement in direct care, dialogue with patients and families, and bedside clinic teaching.’[19] Bayer et al more recently showed that meaning in work was associated with less burnout by using the Work and Meaning Inventory[15]. This inventory maps closely to those *Areas of Worklife* that strongly connected to patient care. The Inventory’s subcategories include the experience of: work that is important and matters, work that promotes personal growth and understanding, and work that broadly contributes to the greater good. Residents experienced work as important with a significant impact when they had autonomy to make patient care decisions (*Area* of control) and enough time to spend with patients as opposed to clerical work (*Area* of workload). Work promoted personal growth and understanding when the education received was focused on patient care (*Area* of value) and allowed for time to foster relationships with patients (*Area* of workload). Finally, residents reported their work contributed to the greater good when they described the intrinsic reward of helping sick children (*Area* of reward). We posit that the interaction and proximity to patients (or lack thereof) may have the largest effect on burnout since a mismatch here influences multiple *Areas of Worklife* and directly affects meaning in work. Especially now, in a time of EMR[20], masking[21], and pandemic related changes in mental health among providers[22], training programs must emphasize the why behind training and medicine to foster meaning in their work.

Limitations to this study include the later addition of questions regarding fairness and workload and the previous role of the PI as chief resident. Although fairness and workload were not initially included, they were iteratively explored after interviews demonstrated that need. Themes were rapidly identified, as responses were significantly consistent with less variability. Second, the PI served as the chief resident for all participants with the exception of the interns during the year prior to the study. While some participants may not have been forthcoming in their answers, on the whole, the PI’s chief resident role and knowledge of the program established a rapport with residents that allowed solicitation of richer information. Additionally, the PI was not involved in resident evaluations at the time of the study. Lastly, all training levels did not have even representation despite equal requests for participation. Nonetheless, third-year resident’s increased participation may be attributable to their time and investment with the residency program. Their senior perspectives likely contributed a more global view.

In sum, applying Leiter and Maslach’s proposed organizational context for burnout to the residency environment yields a comprehensive framework for the identification of potential mismatches between residents and their training environment that may affect burnout. Addressing these specific mismatches may increase meaning in work, which lessens burnout. While many burnout interventions have been studied, the complex and multifaceted syndrome of burnout requires a specific roadmap that focuses on areas to target, and how[4]. Building on previous work identifying the etiology of burnout, this study provides resident perceptions of the individual *Areas of Worklife* to offer programs a practical and focused approach in their battle against burnout. This novel and systematic way of approaching burnout in residency by understanding the meaning and relationships of Maslach’s 6 *Areas of Worklife* in the context of residency is a critical first step to building interventions that successfully address burnout in graduate medical education.

## Supplementary Material

Supplemental Material
