# Supplementary material for: Understanding burnout in Pediatric residency through the lens of the ‘Areas of worklife’
Source: Med Educ Online. 2022 Dec 6;28(1):2152495. doi: 10.1080/10872981.2022.2152495 (PMC9731579; doi:10.1080/10872981.2022.2152495)
Supplement: Supplemental Material [file ZMEO_A_2152495_SM4028.docx]

Supplement: Interview Guide

Control: Can you describe what it means to you to have control over your work in residency? How do you think this can be better accomplished in the residency program?

Values: Can you describe what you value as part of your residency training? How do you think we can improve alignment between resident values and hospital/program values?

Reward: Can you describe what being rewarded in your residency program means to you? How can the program/institution better recognize the contributions of residents?

Community: Can you describe the relationships between residents and other members of the treatment team (nurses, allied health professionals, attendings, other residents) and with program leadership? What changes could be made to improve these relationships?

Fairness: Can you describe what having fairness in your work means to you? How can we improve this?

Workload: Can you describe your workload in residency? What is more taxing, the intensity or the hours worked? How can we improve this?

Are you receiving the things you hoped you would out of your residency program? Please elaborate.
